# Effectiveness Assessment of a Modified Preservation Solution Containing Thyrotropin or Follitropin Based on Biochemical Analysis in Perfundates and Homogenates of Isolated Porcine Kidneys after Static Cold Storage

**DOI:** 10.3390/ijms22168360

**Published:** 2021-08-04

**Authors:** Aneta Ostróżka-Cieślik, Barbara Dolińska, Florian Ryszka

**Affiliations:** 1 Department of Pharmaceutical Technology, Faculty of Pharmaceutical Sciences in Sosnowiec, Medical University of Silesia, Kasztanowa 3, 41-200 Sosnowiec, Poland; bdolinska@sum.edu.pl; 2 “Biochefa” Pharmaceutical Research and Production Plant, Kasztanowa 3, 41-200 Sosnowiec, Poland; f.ryszka@biochefa.pl

**Keywords:** thyrotropin, follitropin, Biolasol, kidneys, pigs, static cold storage

## Abstract

In this paper, we assess the nephroprotective effects of thyrotropin and follitropin during ischaemia. The studies were performed in vitro in a model of isolated porcine kidneys stored in Biolasol (FZNP, Biochefa, Sosnowiec, Poland) and modified Biolasol (TSH: 1 µg/L; FSH 1 µg/L). We used the static cold storage method. The study was carried out based on 30 kidneys. The kidneys were placed in 500 mL of preservation solution chilled to 4 °C. The samples for biochemical tests were collected during the first kidney perfusion (after 2 h of storage) and during the second perfusion (after 48 h of storage). The results of ALT, AST, and LDH activities confirm the effectiveness of Biolasol + p-TSH in maintaining the structural integrity of renal cell membranes. Significantly reduced biochemical parameters of kidney function, i.e., creatinine and protein concentrations were also observed after 48 h storage. The protective effect of Biasol + p-TSH is most pronounced after 2 h of storage, suggesting a mild course of damage thereafter. A mild deterioration of renal function was observed after 48 h. The results of our analyses did not show any protective effect of Biolasol + p-FSH on the kidneys during ischaemia.

## 1. Introduction

One of the challenges in modern transplant technology is the increasing volume of organ donations. Owing to advances in transplant medicine, it has become increasingly possible to successfully transplant organs from older donors, who are often burdened with comorbidities. Searching for solutions that will improve the quality of marginal organs is now considered a priority. The methods of storage before transplantation are of great importance for ensuring the good functional quality of grafts.

The mechanism of ischaemic kidney injury is complex, as discussed earlier [1], and is based on many biochemical processes. The scope of the resulting changes is an indicator of the proper functioning of the organ after transplantation and prognosis in the patient. Disruption of blood supply to the kidneys causes oxygen deficiency in the tissues and the depletion of ATP (adenosine triphosphate) and its substrates. Membrane pumps (ATPases) become ineffective. Mitochondria are damaged. They become swollen and the high-permeability channels in their inner membrane are activated. The structures and functions of the cell membrane are damaged. Free oxygen radicals (ROS) are generated. Cytokines and inflammatory mediators are released. A properly selected composition of preservation solutions enables us to activate repair mechanisms that minimize damage to molecules and/or cell structures and improve the biochemical functions of stored kidneys. Many studies conducted to date indicate that supplementing preservation solutions with hormones may increase the survival rate of grafts [1]. This is due to the direct influence of hormones on organ metabolism and function.

Existing standards for organ procurement and transplantation recommend storing grafts at a reduced temperature using a preservation solution. Simple hypothermic organ preservation is a procedure that can be used on almost all parenchymal organs. This method is used for heart, kidney, liver, lung, and pancreas transplants. It is easy to use, cheap, and effective. Simple hypothermia consists of rinsing the blood off the veins with a cold preservation solution (temperature of 4 °C), which prevents the blood from clotting in the organ and slows down metabolism within the cell, tissue, and organ. Organ cooling reduces the degradation of tissues, reduces the rate of enzymatic reactions, and slows down the consumption of ATP resources. After rinsing with a cold solution, the organs are placed in a double bag, then placed in a transport container filled with ice following the principles of asepsis. Thus secured, they are sent to the transplant centre of the recipient [1,2].

We assessed the nephroprotective effects of thyrotropin (TSH) and follitropin (FSH), which have a similar molecular structure (Table 1). Thyrotropin (TSH, thyroid-stimulating hormone) is secreted by thyrotrophs in the anterior pituitary gland and, in small amounts, in the monocytes of the immune system. It is a heterodimeric glycoprotein hormone (GPH), composed of an α-subunit (with the same amino acid structure as the α-subunit of FSH), and a β-subunit (which is specific to TSH). The α-subunit gene is located on chromosome 6, and the β-subunit gene on chromosome 1. TSH stimulates the secretion of thyroxine and triiodothyronine on the basis of negative feedback in the hypothalamus–pituitary– thyroid axis. In the immune system, it has an immunostimulating effect (increasing the release of interleukin 2 by T lymphocytes). TSH receptors belong to the membrane receptors associated with regulatory G proteins. They are expressed on the surface of thyroid follicular cells, bind thyrotropin by activating adenylate cyclase, and mediate the phosphatidylinositol pathway, leading to the production of thyroid hormones [3,4,5]. Sellitti et al. confirmed that kidneys are also capable of expressing genes for TSHR, and the activity of thyrotropin may serve as a marker of nephron function [6]. Thyroid hormones have a direct impact on the function and hemodynamic parameters of kidneys and the cardiovascular system [7,8]. In a study by Asvold et al., low TSH levels correlated with a reduced glomerular filtration rate (GFR) [9]. Thyrotropin also influences renal plasma flow (RPF) and renal blood flow (RBF) [10]. Kidneys play an important role in the clearance of TSH. Their failure limits the possibility of its excretion [11].

Follitropin (FSH, follicle-stimulating hormone) is a gonadotropin secreted by basophils in the anterior pituitary gland and composed of two subunits, α and β (specific to gonadotropins). The ovaries and testes are the target organs for FSH action. FSH secretion is based on feedback via the hypothalamus–pituitary–gonad axis and is regulated by oestradiol and gonadoliberin (GnRH). FSH interacts with oestrogens to initiate the formation of FSHRs [12,13]. The FSH receptor (like TSH) belongs to the group of endothelial proteins and acts through G proteins, within which the 7TM family, characterized by the presence of seven transmembrane segments, has been distinguished [14,15]. Binding of FSH to the extracellular domain of the receptor mainly stimulates the Gαs/cAMP/protein kinase A (PKA) signalling pathway [16]. Studies by Schanke et al. have shown that follitropin accumulates mainly in the ovaries (granulosa cells) and kidneys (kidney cortex, proximal convoluted tubules of the nephron) [17]. Zhang et al. have confirmed the expression of FSHR on renal tubular epithelial cells (RECs) and on endothelial cells of blood vessels [18]. A strong negative correlation has been demonstrated between eGFR (estimated glomerular filtration rate) and FSH activity in renal function tests [19]. The kidneys play an important role in the excretion of FSH [17].

**Table 1:** Comparison of the structure and function of TSH and FSH.

Hormone	TSH Thyroid-Stimulating Hormone	FSH Follicle-Stimulating Hormone	References
Chemical class	Glycoprotein	Glycoprotein	[20,21]
Source	Anterior pituitary	Anterior pituitary	[20,21]
Cell type	Thyrotrope	Gonadotrope	[20,21]
Mechanism of hormone action	Cyclic AMP	Cyclic AMP	[20,22]
Mechanisms of release	released in a pulsatile fashion	released in a pulsatile fashion	[20,22]
Plasma/serum Concentration	0.4–4.0 mU/L	male: 0.2–4.1 mIU/mL, female: 0.2–14.2 mIU/mL	[23,24]
Molecular weight	28–30 kDa	34 kDa	[25,26]
Circulating half-life	~55 min	3–4 h	[27,28]
Subunits	α/β	α/β	[25,29,30,31,32,33,34]
	TSHα chain consists of 92 amino acids (10.3 kDa)	FSHα chain consists of 92 amino acids (10.3 kDa)	
	TSHβ chain consists of 118 amino acids (13.5 kDa) and forms six intrachain disulphide bonds	FSHβ chain consists of 111 amino acids (12.5 kDa) and forms six intrachain disulphide bridges	
	TSHβ subunit has one an N-glycosylation site at asparagine 23	FSHβ subunit has two an N- glycosylation sites at asparagine 7 and 24	
Principal action	TSH stimulates both synthesis and secretion of thyroid hormones from the thyroid gland; regulates iodide transport into the thyroid; stimulating the Na^+^/I^−^ symporter (NIS) transcription	FSH stimulates ovarian estradiol production and growth of ovarian follicles; is essential for Sertoli cell proliferation and maintenance of sperm quality in the male testis.	[35,36,37,38,39]
Receptor	Membrane–7TM TSHR	Membrane–7TM FSHR	[40,41]
Distribution	Anterior pituitary gland, brain, pars tuberalis, bone, orbital preadipocytes and fibroblasts, kidney, ovary and testis, skin and hair follicles, heart, adipose tissue, hematopoietic and immune cells	Sertoli cells in the testis, granulosa cells in the ovary, uterus, prostate, bone, ovarian surface epithelia, umbilical vein, vessel smooth muscle cells, placenta and placental endothelial cells, fallopian tube, myometrium, endometrial stromal cells, endometrial glandular epithelium, bone, osteoclasts, monocytes, kidney tubules, Purkinje cells, cerebellar medulla, alveolar cells, and hepatocytes	[42,43,44,45,46]
Physiological effects on the organs	Immunotherapeutic, regulates metabolic and inflammatory processes	Regulator for lipogenesis, inflammation, and metabolic disorders	[34,47]

7TM: 7-transmembrane.

The aim of our research was to determine the protective potential of TSH and FSH against ischaemic nephrocytes. The studies were performed in vitro in a model of isolated porcine kidneys stored in Biolasol and modified Biolasol. The effect of follitropin on renal ischaemia–reperfusion injury has not yet been studied.

## 2. Results

**Figure 1:**
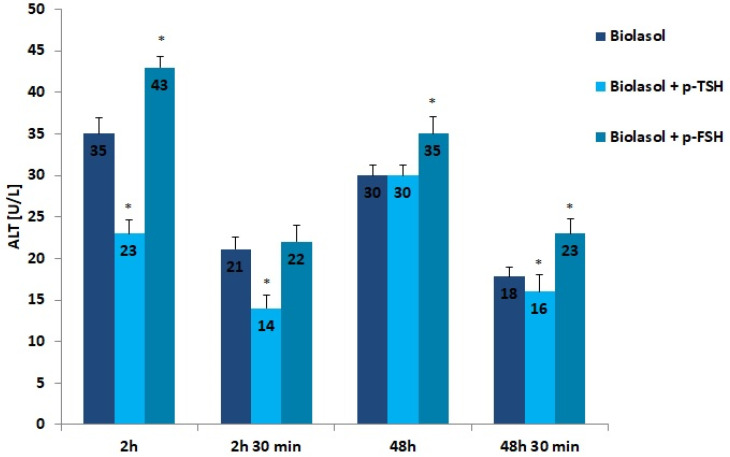
ALT activity in the collected perfusates. The values are expressed as mean ± SEM. * *p* < 0.05 compared to the control group (Biolasol).

**Figure 2:**
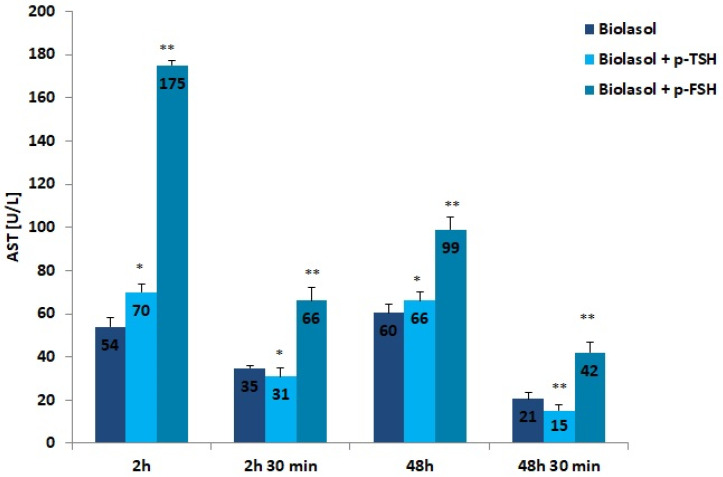
AST activity in the collected perfusates. The values are expressed as mean ± SEM. * *p* < 0.05; ** *p* < 0.01 compared to the control group (Biolasol).

**Figure 3:**
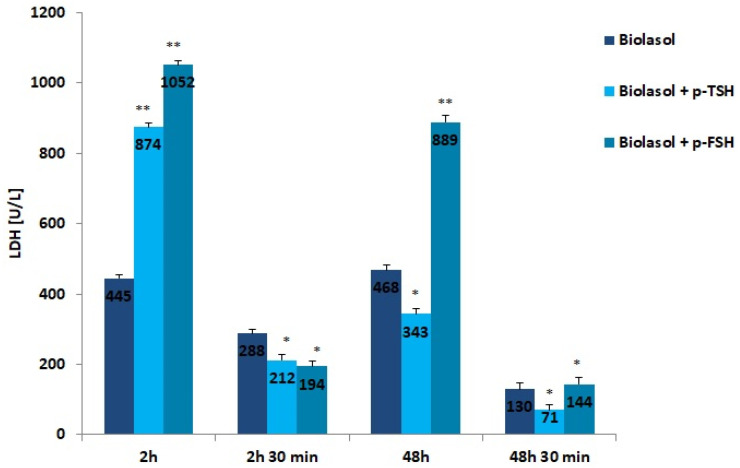
LDH activity in the collected perfusates. The values are expressed as mean ± SEM. * *p* < 0.05; ** *p* < 0.01 compared to the control group (Biolasol).

**Figure 4:**
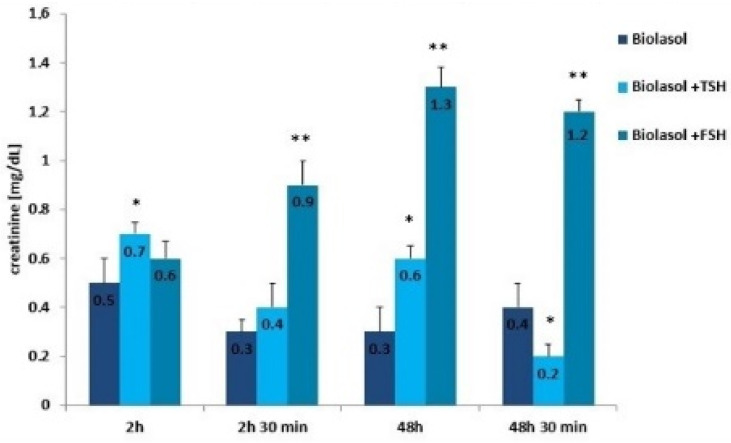
Creatinine concentrations in the collected perfusates. The values are expressed as mean ± SEM. * *p* < 0.05; ** *p* < 0.01 compared to the control group (Biolasol).

**Figure 5:**
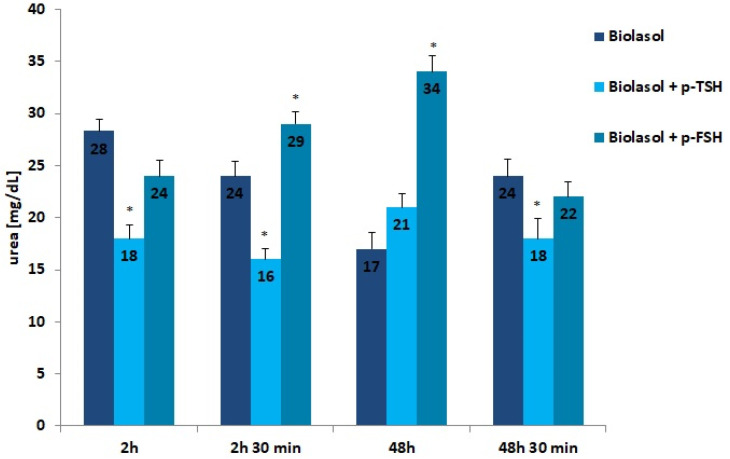
Creatinine concentrations in the collected perfusates. The values are expressed as mean ± SEM. * *p* < 0.05 compared to the control group (Biolasol).

**Figure 6:**
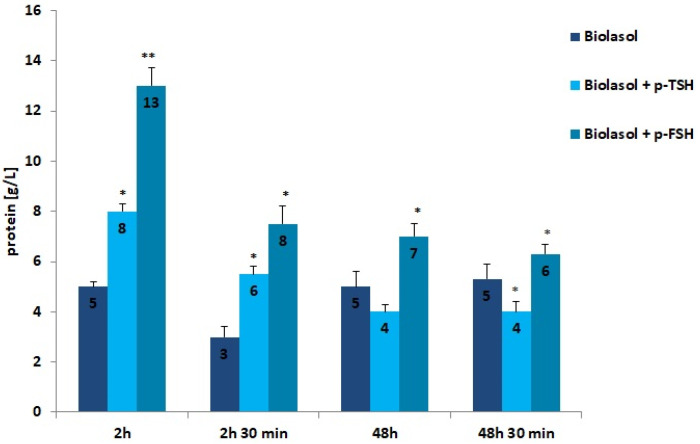
Protein concentrations in the collected perfusates. The values are expressed as mean ± SEM. * *p* < 0.05; ** *p* < 0.01 compared to the control group (Biolasol).

**Figure 7:**
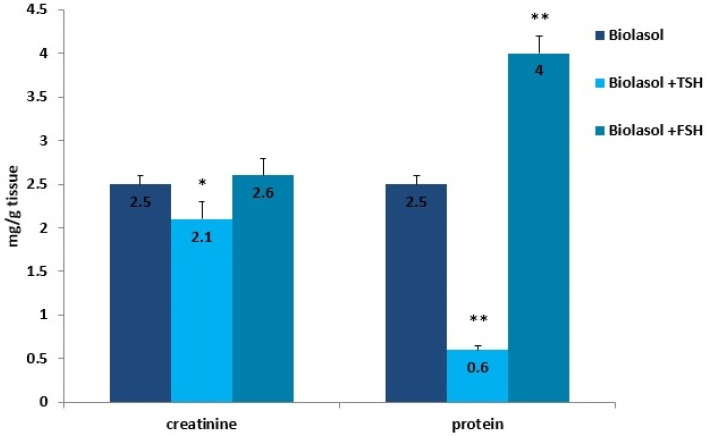
Creatinine and protein concentrations in the kidney homogenates. The values are expressed as mean ± SEM. * *p* < 0.05; ** *p* < 0.01 compared to the control group (Biolasol).

The results of the biochemical tests performed in the control and study groups are presented in Figure 1, Figure 2, Figure 3, Figure 4, Figure 5, Figure 6 and Figure 7. The indirect method, i.e., determination of the activity of enzymes ALT, AST, and LDH, was used to diagnose the degree of damage to kidney cells, which intensifies during ischaemia [48,49,50,51]. The selected markers are sensitive indicators of damage to the structural integrity of the graft. When an organ is damaged, the enzymes move to the outside of the cell. After 48 h graft storage (vs. 2 h), a decrease in ALT activity was observed. The dynamics of this parameter decrease was the highest in the Biolasol + p-FSH group and amounted to 18%. ALT activities in groups A/48 h and B1/48 h were comparable. In turn, the lowest ALT values after 48 h and 30 min were found in the perfusates from the kidneys rinsed with Biolasol + p-TSH. ALT activity was lower in the perfusates from group B1 by 11% vs. group A and by 30% vs. group B2 (*p* < 0.05), indicating the least damage to cell membranes. After 2 h and 30 min as well as 48 h and 30 min, the activity of alanine aminotransferase in the Biolasol + p-FSH group was comparable and not statistically significant, which may indicate ongoing damage to cell membranes.

The addition of hormones to the Biolasol solution also decreased AST activity after 48 h of kidney storage (vs. 2 h). In the group of grafts rinsed with Biolasol + p-TSH, the activity of this enzyme decreased by 6%, whereas in the Biolasol + p-FSH group it decreased by 43%, which was statistically significant (*p* < 0.05; *p* < 0.01). Its activity clearly decreased after perfusion (48 h and 30 min), by 77% in group B1 and by 58% in group B2, and the differences were statistically significant at *p* < 0.01. The highest activity of AST (at all tested time points) was found in perfusates collected after rinsing the kidneys with Biolasol + p-FSH. The probability of damaging the mitochondrial membranes in this group is highest. It should be emphasized that the use of Biolasol + p-TSH fluid reduced the activity of aspartate aminotransferase after 48 h and 30 min by 29% vs. Biolasol and 64% vs. Biolasol + p-FSH, which may prove its effectiveness at reducing nephrocyte damage (*p* < 0.01).

LDH is a cytoplasmic enzyme that converts pyruvate to lactate under conditions of oxygen deficiency. The use of the modified solutions resulted in a decrease in LDH activity after 48 h of kidney preservation. In the Biolasol + p-TSH/48 h group, there was a 61% decrease in LDH activity vs. Biolasol + p-TSH/2 h (*p* < 0.01). In turn, in the Biolasol + p-FSH/48 h vs. Biolasol + p-FSH/2 h model, the decrease in activity was 15% (*p* < 0.05). The lowest activity of this parameter was found in the Biolasol + p-TSH perfusates/48 h 30 min. Low LDH activity indicates that the addition of TSH to the Biolasol fluid at a dose of 1 µg/L protects the kidneys against disturbances in cellular respiration of nephrocytes. TSH has a positive effect on the course of mitochondrial respiration after 48 h of storing ischaemic kidneys.

The activities of ALT, AST, and LDH determined in the Biolasol + p-FSH group after 2-h kidney preservation are noteworthy. The biochemical activities of the above markers were increased and statistically significantly higher than in the control/A group (ALT: 23%; AST: 224%; LDH: 136%). Including FSH in the composition of the Biolasol solution does not prevent kidney damage in the initial storage period. This strategy also shows little effectiveness after 48 h. This may result in a lack of secretory function of the kidneys after transplantation.

Creatinine and urea are classic markers of kidney function. Their measurement allows for the detection of early metabolic changes resulting from ischaemic kidney damage [52]. Kidney damage is accompanied by an increase in creatinine levels, which may correlate with a decrease in renal glomerular filtration. The creatinine concentration increased statistically significantly in the Biolasol + p-FSH perfusates (48 h vs. 2 h) by 116% (*p* < 0.01) and slightly decreased after 48 h 30 min (statistically insignificantly). This is evidence of a persistent deterioration in renal function and a decreasing number of active nephrons. In turn, in the Biolasol + p-TSH group, the creatinine concentration decreased (48 h vs. 2 h), but the difference was not statistically significant. A statistically significant decrease in creatinine activity in the B1 model group was found after 48 h 30 min of perfusion (67%, *p* < 0.01).

Urea is a product of protein nitrogen metabolism. It is excreted by the kidneys by glomerular filtration and reabsorption in the proximal tubule and partially in the distal tubule. Urea concentration increases with a decrease in renal glomerular filtration. Rinsing the kidneys with Biolasol + p-FSH resulted in a 42% increase in the urea concentration over 48 h (vs. 2 h) and its decrease by 35% after 48 h 30 min of perfusion (both differences were statistically significant, *p* < 0.05). The differences in the urea levels determined in the Biolasol + p-TSH group at all time points were not statistically significant.

Low molecular weight proteins and albumin is reabsorbed in the proximal coils. Injury of tubulointerstitial tissue leads to the loss of filtered proteins and reabsorbable proteins. Proteins are also released by damaged renal tubular epithelial cells. When the glomeruli are damaged, the permeability of the capillaries to proteins increases, especially albumin [53]. The use of the Biolasol + p-TSH and Biolasol + p-FSH solutions resulted in an increase in the protein concentration in the perfusates after 2 h of kidney storage compared to the control/A solution by 60% (*p* < 0.05) and 160% (*p* < 0.01), respectively. The protein concentration decreased after 48 h of graft storage (vs. 2 h) by 46% in group B2, and by 50% in group B1 (both differences were statistically significant at *p* < 0.05). This level was maintained for 48 h 30 min (the difference in the B2 group was statistically insignificant). Rinsing the kidneys with Biolasol + p-FSH probably causes damage to the glomerular membrane.

Kidney storage in Biolasol + p-TSH led to a decrease in the creatinine concentration in the homogenates by 16% (*p* < 0.05) compared to the values observed in the homogenates obtained after perfusion with Biolasol. Protein retention was greatest in ischaemic kidney homogenates flushed with Biolasol + p-FSH cold fluid. The use of solution B1 also caused a significant decrease in the protein concentration in the homogenates (76%, *p* < 0.01) vs. solution A. An increase in the protein concentration (vs. A) was found in the kidney homogenates stored in Biolasol + p-FSH (60%, *p* < 0.01). An excessive amount of protein enhances the expression of cytokines responsible for the development of inflammation and gradual fibrosis of the interstitial tissue.

## 3. Discussion

Organ preservation solutions are designed to prevent the consequences of ischaemia–reperfusion injury and the effects of low temperatures. The ideal solution for organ storage should slow down cellular metabolism, contain substrates allowing for the regeneration of high-energy phosphate compounds (ATP), keep the biochemical parameters of cells as unchanged as possible, reduce the formation of free radicals and peroxides, reduce cell swelling by stabilizing the cell membrane, ensure adequate oncotic pressure, and minimize acidosis [1].

We investigated the use of Biolasol for kidney storage in this research. It is the first and only solution developed in Poland intended for organ perfusion and preservation. It provides better acid–base homeostasis for isolated porcine kidneys. It stabilizes the tissue and slows down the rate of changes that occur during organ storage [54,55]. Kidney transplantation with the use of Biolasol fluid indicates its nephroprotective properties. In a study by Jozwik et al., 42 kidneys were preserved by cold storage. The incidence of delayed graft function (DGF) episodes was assessed using Biolasol and UW solutions. The number of DGF episodes was comparable (38% vs. 33%; *p* = ns) [56]. In our previous studies, we used Biolasol as a base to evaluate the effectiveness of pharmacological substances in flushing and preserving grafts. We have shown that prolactin (PRL, dose: 1 µg/L) [51,57], lutropin (LH, dose: 0.01 µg/L) [58], and selenium (Se^4+^, dose: 1 µg/L) [48] have a beneficial protective effect on the kidneys. The addition of Zinc to the Biolasol composition at a dose of 1 µg/L showed minor effectiveness in the protection of nephrons [59].

Thyroid hormones regulate the proper functioning of the kidneys and, in particular, the glomerular filtration rate (GFR) [60]. Junik et al. [61] found that thyroid dysfunction occurs in patients after allogeneic kidney transplantation. The transplant affects the metabolism of thyroid hormones, causing, inter alia, a decrease in TSH levels in the early postoperative period. This may be due to stress induced by surgery [62] or immunological conditions after transplant [63].

The present research results indicate that TSH and FSH introduced into the composition of Biolasol affect the efficiency of the storage of isolated porcine kidneys. The protective effect on ischaemic nephrocytes is shown by Biolasol + p-TSH, in which the dose of the hormone was 1 µg/L. The results of ALT, AST, and LDH activities confirm its effectiveness in maintaining the structural integrity of renal cell membranes [51]. Significantly reduced biochemical parameters of kidney function, i.e., urea, creatinine, and protein concentrations, were also observed (vs. Biolasol + p-FSH) after 48 h storage. In clinical practice, a significant increase in the creatinine concentration in the blood serum determines the 50% loss of normal glomerular functions [64]. Urea is an indicator of the intensity of protein breakdown. Its increase correlates with decreased renal perfusion [65]. In turn, an increase in protein indicates a deterioration in kidney function.

Similar research results were obtained by Caban et al., who used a HTK + p-TSH (TSH: 1 µg/L) solution to store isolated porcine kidneys using the SCS method [66]. TSH had a cytoprotective effect on the grafts during their 24 and 48 h storage. The addition of the hormone compensates for metabolic disorders in ischaemic kidneys and maintains the energy potential of cells [66]. TSH indirectly controls the rate of cellular metabolism, the intensification of catabolic processes, and the energy expenditure in cells. It affects the mitochondria and cellular organelles responsible for ATP production. However, the mechanism of action is unknown [67]. TSH modulates immune function by stimulating thyroid hormones. It also acts on lymphoid cells [68]. The protective effect of TSH on kidneys may also result from TSHR expression on some cells of the immune system [69]. TSH receptors are expressed in dendritic cells and in CD45RBhigh lymph node T cells [70]. Some authors have suggested that TSH can be produced directly by human monocytic cells [71].

It was found that TSH is involved in the regulation of vascular homeostasis [72,73]. Donini et al. [74] analysed its influence on human aortic endothelial cells. They found that TSH induced an increase in cAMP (cyclic AMP) and nitric oxide concentration, and a decrease in endothelin and tissue plasminogen activator secretion. Clinical studies conducted by Napoli et al. [75] confirm that rhTSH enhances endothelial function in the resistance vessels.

The efficacy of FSH as a component of preservation solutions has not been the subject of much research so far. The results of our analyses did not show any protective effect of Biolasol + p-FSH (p-FSH: 1 µg/L) on kidneys during ischaemia. The increased activities of ALT, AST, and LDH in the Biolasol + p-FSH group after 2 h of kidney preservation are noteworthy. This probably indicates increased FSHR expression on renal tubular epithelial cells (RECs), which may result in tubulointerstitial fibrosis [19]. Zhang et al., using an ovariectomized mouse model, found that the administration of recombinant FSH at a dose ranging from 0.15 IU to 0.3 IU increases the protein, creatinine, and urea concentrations in the blood serum of the rodents. Therapy with FSH contributes to renal dysfunction via the AKT/GSK-3β/β-catenin pathway in HK-2 cells [19]. It has been found that an increase in FSH concentration also promotes the expression of M-CSFR (macrophage-colony stimulating factor) [76]. Chronic activation of monocytes can cause metabolic, haematological, and immunological disturbances in patients with chronic renal failure [77]. Moreover, M-CSF activates the proliferation of pro-inflammatory macrophages [78]. Some authors believe that M-CSF mediates the regeneration of the renal tubular epithelium after acute renal injury [79]. We suggest that further research needs to be conducted on the effect of FSH (as a component of preservation solutions) on nephrocyte function during ischaemia, taking into account the dose of the hormone.

Kidney ischaemic injury is a complex process that leads to changes in graft structure and function. The protective effect of Biasol + p-TSH is most pronounced after 2 h of storage, suggesting a mild course of damage thereafter. A mild deterioration of renal function was observed after 48 h. The results of our analyses did not show any protective effect of Biolasol + p-FSH on the kidneys during ischaemia.

## 4. Materials and Methods

### 4.1. Preservation Solution

This study used Biolasol solution (FZNP, Biochefa, Sosnowiec, Poland). The composition and functions of its individual components are presented in Table 2. The hormones, i.e., porcine thyrotropin (TSH) and porcine follitropin (FSH), were from FZNP. The hormones were added to the Biolasol solution immediately prior to the experiment. All the substances used in the study were of analytical grade.

### 4.2. Animals

The study was carried out based on 30 isolated porcine kidneys, which came from 15 Polish Large White pigs. The weight of animals ranged from 90 to 110 kg, age: 175–180 days. The pigs were slaughtered at the Meat Plant H.A.M in Radzionków (Poland) in a separate room using a voltage of 220 V. Subsequently, both kidneys were excised for examination. The project was approved by the II Local Ethics Commission for Animal Experiments in Cracow (No. 1046/2013, approval date: 4 June 2013) and was carried out in accordance with the European Union regulations (Directive 86/609 CEE) on the protection of animals during slaughter.

### 4.3. Study Groups of Animals

Thirty kidneys used for the study were divided into three groups and preserved by static cold storage (a process whereby the preservation solution is infused into the organ and then stored statically at hypothermic temperatures):

Group A (control). Biolasol; static cold storage (*n* = 10 kidneys) for 2 h and 48 h;
Group B1. Biolasol + p-TSH (1 µg/L); static cold storage (*n* = 10 kidneys) for 2 h and 48 h;
Group B2. Biolasol + p-FSH (1 µg/L); static cold storage (*n* = 10 kidneys) for 2 h and 48 h;

### 4.4. Experimental Protocol

**Figure 8:**
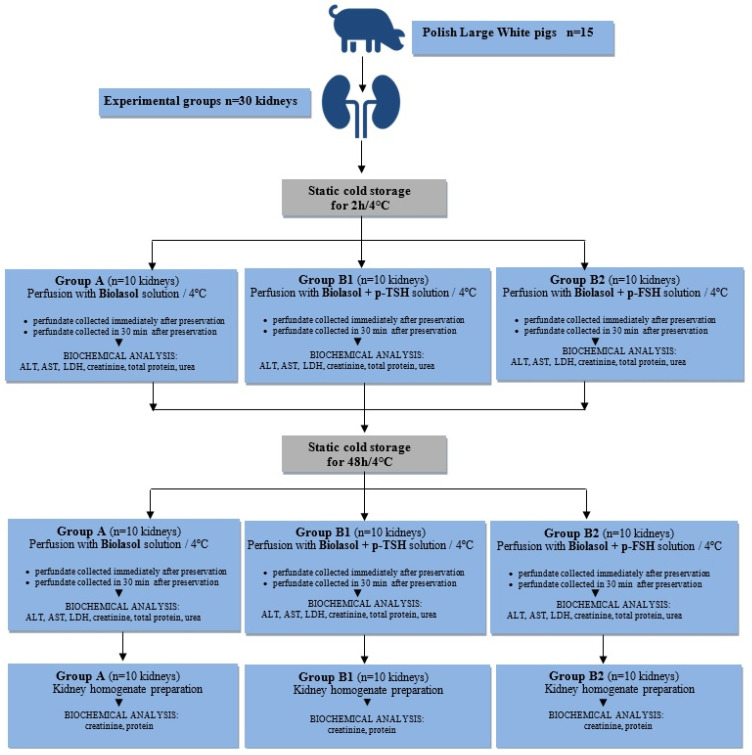
Study design.

The kidneys were harvested according to the procedures described in our previous work [48]. The harvested organs were prepared on the “side table”. The vasculature was rinsed out of the blood and filled with preservation solution. The isolated porcine kidneys were placed in 0.5 L of Biolasol (Group A) or modified Biolasol (Groups B1 and B2) chilled to 4 °C. The grafts were transported in thermostatic ice containers (temperature of 4–6 °C) to the Biochefa FZNP laboratory and stored by static cold storage (SCS) for 2 h. After this time, the renal artery was cannulated (Nela-ton CH08 catheter, ConvaTec, Deeside, UK) and perfused (pressure of 73.5 mmHg H_2_O) ensuring a continuous flow of solution stream. The samples for biochemical tests (10 mL volume) were collected during the first kidney perfusion, after 2 h of storage (at *t* = 0 and *t* = 30 min) and during the second perfusion, after 48 h of storage (at *t* = 0 and *t* = 30 min). The collected perfusates were centrifuged (3000 rpm, 15 min, 4 °C), and the collected supernatant was stored at −20 °C until analytical determinations were completed. Diagnostic tests were used to indirectly assess renal function [48,49,50,51]. The graft samples intended for biochemical analysis in kidney homogenates were collected after 48 h 30 min (Figure 8).

### 4.5. Measurements of Clinical Parameters

#### 4.5.1. Determination of Alanine Aminotransferase

The activity of alanine aminotransferase (ALT) was determined with the kinetic method using the bioMérieux diagnostic kit (Lyon, France) according to the manufacturer’s instructions. The absorbance value was read at 340 nm (Marcel S330 spectrophotometer, Warsaw, Poland). The photometric accuracy of the spectrophotometer was ±0.005 Abs. The reference interval for pigs was 31–58 U/L [80].

#### 4.5.2. Determination of Aspartate Aminotransferase

The activity of aspartate aminotransferase (AST) was determined with the kinetic method using a bioMérieux diagnostic kit according to the manufacturer’s instructions. The absorbance value was read at 340 nm (Marcel S330 spectrophotometer). The photometric accuracy of the spectrophotometer was ±0.005 Abs. The reference interval for pigs was 32–84 U/L [80].

#### 4.5.3. Determination of Lactate Dehydrogenase Activity

The activity of lactate dehydrogenase (LDH) was determined with the kinetic method using a bioMérieux diagnostic kit according to the manufacturer’s instructions. The absorbance value was read at 340 nm (Marcel S330 spectrophotometer). The photometric accuracy of the spectrophotometer was ±0.005 Abs. The reference intervals for pigs was 380–634 U/L [80].

#### 4.5.4. Determination of Creatinine Concentration

Creatinine concentration was determined using reagent kits from Pointe Scientific INC (Marseille, France), according to the manufacturer’s instructions. The absorbance value was read at 490 nm (Marcel S330 spectrophotometer). The photometric accuracy of the spectrophotometer was ±0.005 Abs. The reference interval for pigs was 1.0–2.7 mg/dL [80].

#### 4.5.5. Determination of Total Protein Concentration

Total protein concentration was determined using reagent kits from Pointe Scientific INC according to the manufacturer’s instructions. The absorbance value was read at 540 nm (Marcel S330 spectrophotometer). The photometric accuracy of the spectrophotometer was ±0.005 Abs. The reference interval for pigs was 7.9–8.9 g/dL [80].

#### 4.5.6. Determination of Urea Concentration

Urea concentration was determined using reagent kits from Pointe Scientific INC according to the manufacturer’s instructions. The absorbance value was read at 340 nm (Marcel S330 spectrophotometer). The photometric accuracy of the spectrophotometer was ±0.005 Abs. The reference interval for pigs was 10–30 mg/dL [80].

#### 4.5.7. Biochemical Analysis in Kidney Homogenates

Kidney samples were collected after perfusion, after 48 h 30 min. The samples were homogenized in chilled 0.1 M phosphate buffer, pH = 7. The homogenates were centrifuged for 3 min at 15,000 rpm. The creatinine and protein concentrations were determined in the supernatants.

### 4.6. Statistical Analysis

The test results are shown as mean ± SEM. The occurrence of statistically significant differences between the analysed groups was determined using one way analysis of variance (ANOVA). After rejecting the null hypothesis (*p* < 0.05), statistical differences between the groups were compared by post hoc Tukey’s test (*n* = 10 for each group) [81]. The adopted level of statistical significance was *p* < 0.05. Statistica version 13.1 software (StatSoft, Cracow, Poland) was used in the calculations.

## 5. Conclusions

TSH at a dose of 1 µg/L has a protective effect on ischaemic nephrocytes in a model of isolated porcine kidneys stored in a modified Biolasol solution. The effectiveness of FSH at a dose of 1 µg/L is questionable.

## Figures and Tables

**Table 2:** The composition of Biolasol solution.

Component		Function
Potassium chloride	10 mmol/L	Regulates the water–electrolyte balance; source of K^+^
Calcium chloride	0.5 mmol/L	Source of Ca^2+^ and Cl^-^ ions; influences electrolyte balance at the level of extracellular fluid; extracellular Ca^2+^ stabilizes membranes
Fumarate magnesium	5 mmol/L	Protects physicochemical properties of the solution and ensures the stability of other components; as a source of Mg^2+^, magnesium fumarate affects the lipid bilayer integrity and function
Dextran 70	0.7 g/L	It affects maintenance of the correct fluid volume in aqueous areas; it prevents cell oedema; improves capillary circulation, decreases blood cell aggregation
Sodium bicarbonate	5 mmol/L	pH buffer; helps to maintain proper pH through the action of compensation acidic metabolites formed as a result of increased anaerobic metabolism; source of Na^+^ and HCO_3_^–^ for maintaining electrolyte balance
Tri-sodium citrate	30 mmol/L	Anticoagulation factor; impact on acid–base homeostasis, source of Na^+^
Glucose anhydrous	167 mmol/L	Source of energy; regulates fluid distribution in fluid compartments; maintains the osmotic gradient between extra- and intracellular fluids; prevents cell oedema
di-sodium edetate	5 mmol/L	By chelation of calcium ions, it blocks activation of zymogens; removes catalytic effect on catabolic reactions by decreasing free ions level

## Data Availability

Samples of the compounds are available from the authors.
